# Effects of *n*-3 PUFAs on Intestinal Mucosa Innate Immunity and Intestinal Microbiota in Mice after Hemorrhagic Shock Resuscitation

**DOI:** 10.3390/nu8100609

**Published:** 2016-09-29

**Authors:** Feng Tian, Xuejin Gao, Li Zhang, Xinying Wang, Xiao Wan, Tingting Jiang, Chao Wu, Jingcheng Bi, Qiucheng Lei

**Affiliations:** 1 Research Institute of General Surgery, Jinling Hospital, Medical School of Nanjing University, Nanjing 210002, China; tianfeng_nju@163.com (F.T.); xuejingao@163.com (X.G.); zlshe1107@163.com (L.Z.); wanxiao_njzy@163.com (X.W.); jiangtingting08med@163.com (T.J.); wuchao0008@163.com (C.W.); ahbijingcheng@163.com (J.B.); lqiuchenggd@163.com (Q.L.); 2 Department of General Surgery, South Medical University, Guangzhou 510515, China

**Keywords:** *n*-3 PUFAs, hemorrhagic shock, intestinal mucosa, innate immunity, intestinal microbiota, intestinal barrier function

## Abstract

*n*-3 polyunsaturated fatty acids (PUFAs) can improve the function of the intestinal barrier after damage from ischemia-reperfusion or hemorrhagic shock resuscitation (HSR). However, the effects of *n*-3 PUFAs on intestinal microbiota and the innate immunity of the intestinal mucosa after HSR remain unclear. In the present study, 40 C57BL/6J mice were randomly assigned to five groups: control, sham, HSR, HSR + *n*-3 PUFAs and HSR + *n*-6 PUFAs. Mice were sacrificed 12 h after HSR. Liver, spleen, mesenteric lymph nodes and terminal ileal tissues were collected. Intestinal mucosae were scraped aseptically. Compared with the HSR group, the number of goblet cells increased, expression of mucin 2 was restored and disturbed intestinal microbiota were partly stabilized in the PUFA-administered groups, indicating that both *n*-3 and *n*-6 PUFAs reduced overproliferation of Gammaproteobacteria while promoting the growth of Bacteroidetes. Notably, *n*-3 PUFAs had an advantage over *n*-6 PUFAs in improving ileal tissue levels of lysozyme after HSR. Thus, PUFAs, especially *n*-3 PUFAs, partly improved the innate immunity of intestinal mucosa in mice after HSR. These findings suggest a clinical rationale for providing *n*-3 PUFAs to patients recovering from ischemia-reperfusion.

## 1. Introduction

One of the features of the intestinal barrier, besides the capacity to absorb nutrients, is the ability to recover from damage caused by gut pathogens. The intestinal barrier also prevents the commensal organisms from entering the circulation. Surgical patients often experience ischemia-reperfusion injury (IRI) because of trauma, severe blood loss and septic shock [1]. The intestinal mucosa is one of the tissues most frequently affected by IRI [2,3], resulting in impaired intestinal barrier function and triggering a series of inflammatory reactions. Given the association between IRI and poor clinical outcomes, treatment of IRI is a major challenge for surgeons [4]. In previous studies, various models that completely blocked regional intestinal blood flow were used to study intestinal IRI [5,6,7]. However, the most common causes of intestinal IRI in surgical patients and those suffering from trauma are systemic hypovolemia and hypoperfusion [1]. The controlled hemorrhagic shock resuscitation (HSR) mouse model can be used to study the effect of intestinal IRI on intestinal barrier function caused by systemic hypovolemia [6,8]. Previous studies have shown that systemic and regional intestinal IRI can cause similar damage to the intestinal mechanical barrier, including destruction of tight junctions [6,7]. As a result, the following intestinal bacterial translocation can induce further injury in distant tissues. Furthermore, intestinal mucosa innate immunity (IMII) also plays important roles in removing pathogenic bacteria and alleviating intestinal injury [9,10]. Antimicrobial peptides and mucins secreted by Paneth and goblet cells, respectively, are involved in IMII [10,11]. IMII contributes to the homeostasis of the gut microbiome composition, which in turn benefits the intestinal mucosa by preventing overgrowth and invasion of pathogenic bacteria [10,12]. Previous studies have shown that localized intestinal IRI could impair the function of both Paneth and goblet cells [11,13], but the effect of IRI caused by systemic hypovolemia is still unclear.

Previous studies indicated that *n*-3 polyunsaturated fatty acids (PUFAs) could reduce the levels of pro-inflammatory cytokines, regulate immune function, protect vital organs and enhance clinical outcomes [14,15,16,17]. The routine use of *n*-3 PUFAs in surgical intensive care units for postoperative patients is recommended by the latest guidelines of the American Society for Parenteral and Enteral Nutrition because of their significant anti-inflammatory effects [18]. *n*-3 PUFAs have been reported to protect the intestinal barrier from intestinal IRI by modifying the intracellular intestinal fatty acid binding protein/peroxisome proliferator-activated receptor pathway and upregulating tight junction proteins [7]. Another study suggested that *n*-3 PUFAs could also maintain intestinal tight junctions after HSR [6]. However, it remains to be determined whether *n*-3 PUFAs could also improve the functions of Paneth and goblet cells and maintain gut microbiome composition after HSR.

Here, we used a mouse model of HSR to simulate systemic hypovolemia and to determine whether *n*-3 PUFAs could improve the function of Paneth and goblet cells upon HSR-induced damage to IMII. We speculated that administration of *n*-3 PUFAs could also prevent the disturbance of the intestinal microbiota during HSR. We believe that our findings can provide insights into the mechanisms underlying the protective effects of *n*-3 PUFAs in the intestinal barrier after IRI.

## 2. Materials and Methods

### 2.1. Animals

Animal protocols were approved by the model animal research center (MARC) and the Jinling Hospital of Nanjing University, Nanjing (MARC XG67). Male 10–12-week-old C57BL/6J mice were provided by the MARC at Nanjing University. The animals were kept under pathogen-free conditions at ambient temperature (20–26 °C) and 40%–70% humidity, with a 12-h light/dark cycle and free access to food and water. The mice were allowed to acclimatize to the new environment for at least 7 days prior to manipulation.

### 2.2. Study Design

In total, 24 C57BL/6J mice were randomly assigned to 3 groups (*n* = 8 per group): (1) control (CON); (2) *n*-3 PUFAs (*n*-3 PUFAs); and (3) *n*-6 PUFAs (*n*-6 PUFAs). During the 3 days preceding surgery, mice in the *n*-3 PUFA and *n*-6 PUFA groups were administered daily doses of 0.2 g/kg body weight of *n*-3 PUFAs (Omegaven^®^ 10%, Huarui Pharmaceutical Co., Ltd., Wuxi, China) and *n*-6 PUFAs (Intralipid^®^ 10%, Huarui Pharmaceutical Co., Ltd.), respectively, through the caudal vein. The same volume of saline solution was injected into the caudal vein of mice in the control group. Before operation, mice that received *n*-3 PUFAs, *n*-6 PUFAs and saline solution were sacrificed, and their intestinal tissue was collected to investigate whether the PUFAs changed the IMII prior to HSR.

Another 40 C57BL/6J mice were randomly assigned to 5 groups (*n* = 8 per group): (1) control (CON); (2) sham (Sham); (3) 12 h after HSR (HSR); (4) HSR + *n*-3 PUFAs (*n*-3 PUFAs); and (5) HSR + *n*-6 PUFAs (*n*-6 PUFAs). The mice were anesthetized with 0.15 mL/10 g body weight of 2.5% avertin intraperitoneally and with 5 mg/kg body weight of carprofen subcutaneously. Mice in the sham group were subjected to skin incision and ligation of the right superficial femoral artery. Mice in the HSR, *n*-3 PUFA and *n*-6 PUFA groups had the right superficial femoral artery cannulated with PE10 tubing as described in previous studies [6,8]. The arterial catheter was connected to a pressure monitor (BD6240; Chengdu Instrument Factory, Chengdu, China) to follow mean arterial pressure (MAP). Blood was withdrawn over 15 min via the femoral artery catheter to reduce the MAP to 30 mmHg. Blood was then withdrawn or returned to the animal as required to maintain a MAP of 30 ± 3 mmHg for 90 min. At the end of the shock period, mice were resuscitated with the shed blood plus a two-fold volume of Ringer’s lactate. Next, the catheter was removed; the artery was ligated; and the skin incision was closed under aseptic conditions. After surgery, mice in the HSR, *n*-3 PUFA and *n*-6 PUFA groups received normal saline solution, *n*-3 PUFAs and *n*-6 PUFAs, respectively, as during the preoperative treatment. During surgery and recovery, the mice were placed on a heated platform. When there was any evidence of struggle, shortness of breath or any other reactions during surgery, the mice were anesthetized again with an additional 0.10 mL/10 g body weight of 2.5% avertin intraperitoneally. Medical monitoring was performed throughout recovery from surgery and anesthesia.

Mice were anesthetized as described above and sacrificed 12 h after resuscitation. The liver, spleen and mesenteric lymph nodes were collected aseptically for bacterial culture. Next, the small intestine was removed and flushed with 20 mL of cold calcium- and magnesium-free Hank’s Balanced Salt Solution (CMF-HBSS; Life Technologies, Carlsbad, CA, USA). The mucosa of the terminal ileum was collected aseptically for the investigation of bacterial colonization. Distal ileal tissue samples (2 cm) were collected and stored at −80 °C for subsequent analysis. Another tissue sample was treated with 4% paraformaldehyde at 4 °C overnight and then transferred to 70% ethanol and stored at 4 °C for histopathological analysis.

### 2.3. Bacterial Culture

Collected tissue samples were weighed, and each sample was homogenized aseptically in 9× the tissue weight of phosphate-buffered saline (PBS). Diluted homogenates (100 µL) were plated and grown aerobically on blood agar medium (Difco, BD, Franklin Lakes, NJ, USA) at 37 °C for 48 h. Colony-forming units (CFUs) were counted to determine whether bacterial translocation had occurred in the mice. The result was deemed positive when >100 CFU/g of tissue were found in the cultures.

### 2.4. Enzyme-Linked Immunosorbent Assay

The enzyme-linked immunosorbent assay (ELISA) was used to measure the levels of the cytokines IL-4 and IL-10. Distal ileal tissue was carefully homogenized in cold lysis buffer, and equally concentrated samples were centrifuged at 10,000× *g* for 15 min at 4 °C. The supernatant was extracted and re-centrifuged at 10,000× *g* for 15 min at 4 °C. The concentrations of IL-4 and IL-10 in the supernatant were measured using an ELISA kit (ExCell Bio, Shanghai, China) according to the manufacturer’s protocol. Data were expressed as pg/mg tissue.

### 2.5. Western Blot Analysis of Lysozyme and Mucin 2

Equal amounts of total protein extracted from ileal tissue samples were heated at 95 °C for 5 min and then separated by electrophoresis on 15% (lysozyme, 15 kDa) and 5% (mucin 2, 621.5 kDa) SDS-polyacrylamide gels. Proteins were transferred onto polyvinylidene fluoride (PVDF) membranes, which were washed 5× in PBS with 1% Tween^®^ 20 (PBST), blocked in bovine serum albumin (BSA) for 1 h and then incubated with primary antibodies (rabbit-anti-lysozyme, 1:5000 dilution; mouse-anti-mucin 2, 1:1000 dilution; both Abcam, Cambridge, UK) overnight at 4 °C. Glyceraldehyde-3-phosphate dehydrogenase (GAPDH) was used as an internal control to adjust the density of bands on multiple membranes. Subsequently, the membranes were washed and incubated with the corresponding horseradish peroxidase-linked secondary antibodies for 1 h at 25 °C with constant agitation. After washing, the membranes were incubated with electrochemiluminescence solution for 5 min, and the bands of target proteins were detected using Kodak film. Gray-scale analysis of the bands was performed using ImageJ software. Data were presented as the expression ratios of the target protein relative to GAPDH.

### 2.6. Quantitative Polymerase Chain Reaction Analysis of the Antibacterial Lectin RegIIIγ and Cryptdin 24

Frozen distal ileal tissue samples were ground in the RNAiso Plus extraction reagent (TaKaRa Bio, Tokyo, Japan). RNA was extracted, and its purity and concentration were determined according to the manufacturer’s protocol. Total RNA (1 μg) was used as template for reverse transcription according to the manufacturer’s instructions (TaKaRa Bio). SYBR^®^ Green Select Master Mix (TaKaRa Bio) was used to perform qPCR. Relative mRNA expression levels of RegIIIγ and Cryptdin 24 were calculated based on Ct values. The geometric mean expression of GAPDH was used for normalization. The primers used in this study are described in Table 1.

### 2.7. Immunohistochemistry of Lysozyme in Ileal Tissue Samples

After dehydration, ileal tissue sections were fixed in paraffin wax. The wax blocks were cut into 5-µm-thick slices and deparaffinized. After antigen retrieval (with sodium citrate buffer) and blocking (5% BSA in PBST for 1 h), ileal tissue slices were incubated with an anti-lysozyme antibody (Abcam) overnight at 4 °C. Subsequently, the sections were processed using a 3,3′-diaminobenzidine detection kit (SP-9000-D; ZSGB-Bio, Beijing, China) according to the manufacturer’s instructions. The sections were then counterstained with hematoxylin, and the coverslips were fixed with 50% neutral resins.

### 2.8. Periodic Acid-Schiff Staining

After dehydration through graded ethanol washes, ileal tissue sections were fixed in paraffin wax. The wax blocks were cut into 5 µm-thick slices, deparaffinized, rehydrated (2 × 2 min 100% ethanol, 1 × 2 min 95% ethanol, 1 × 2 min 90% ethanol, 1 × 2 min 80% ethanol, and 1 × 2 min 70% ethanol) and placed into distilled water. Samples were then stained with periodic acid-Schiff stain (Abcam) according to the manufacturer’s protocol. The average number of goblet cells per microscopic field of vision was counted by two independent, blinded researchers (Feng Tian and Xuejin Gao). Three fields were counted for one mouse (original magnification = 40×).

### 2.9. DNA Extraction and 16S rRNA Pyrosequencing

DNA extraction and 16S rRNA pyrosequencing were performed as described in our previous study [19]. Briefly, a terminal ileal tissue sample (~1 cm in length) was flushed gently with 1 mL of CMF-HBSS, and a longitudinal incision was made. The intestinal mucosa was scraped and collected aseptically from the ileum tube. Bacterial DNA was extracted from each sample using the PureLinkTM Genomic DNA Mini Kit (K1820-00; Life Technologies) and stored at −20 °C.

The broadly-conserved primers 517F (5′-GCCAGCAGCCGCGGTAA-3′) and 926R (5′-CCGTCAATTYYTTTRAGTTT-3′) and Platinum TM HiFi Polymerase (Life Technologies) were used to amplify the target region. The PCR amplification product was separated on a 2% agarose gel, and the target DNA fragment was recovered and quantified using a Qubit HS DNA kit (Thermo Fisher Scientific, Waltham, MA, USA). The non-conserved V4–V6 region, comprising approximately 400 base pairs, was selected to construct a community library using the Ion Plus Fragment library kit (Thermo Fisher Scientific). The amplicon libraries were sequenced by the Ion Torrent Personal Genome Machine system using the Ion PGMTM Sequencing 400 Kit (Thermo Fisher Scientific) according to the corresponding protocol. The taxonomic status (phylum and genus) was assigned to each read using a parallelized version of the Greengenes database [20]. The 16S sequencing data are shown in Table S1.

### 2.10. Statistical Analysis

Data counts from different groups were expressed as frequencies. Differences between groups were assessed using the chi-squared or Fisher’s exact probability tests. Measurements from different groups were expressed as the mean ± standard deviation (normally distributed) or median (non-normally distributed). Differences between groups were tested by one-way analysis of variance. Differences between any two means were assessed by least significant difference or Dunnett’s T3 tests. The Kruskal–Wallis test was used if the data were not normally distributed. Statistical calculations were performed using SPSS 19.0 software (SPSS Inc., Chicago, IL, USA).

## 3. Results

### 3.1. Bacterial Culture and Colony Counting

Bacterial translocation occurs once the intestinal barrier function is damaged. Therefore, bacterial culture and colony counting were used to evaluate the severity of damage to intestinal barrier function. Positive rates of bacterial translocation and colony counts in the mesenteric lymph nodes (MLN) were significantly higher in the HSR, *n*-3 PUFA and *n*-6 PUFA groups than in the control or sham groups (*p* < 0.05, respectively) (Table S2 and Table 2). There were fewer bacterial colonies in the *n*-3 PUFA and *n*-6 PUFA groups than in the HSR group (550 CFUs/g or 900 CFUs/g vs. 4000 CFUs/g; *p* < 0.05, respectively) (Table 2). In contrast, the positive rates of bacterial translocation and bacterial colony counts in the liver and spleen did not differ significantly among the five groups because of large intra-group variation (Table S2 and Table 2).

### 3.2. Expression and Localization of Lysozyme in Ileal Tissue

**Figure 1:**
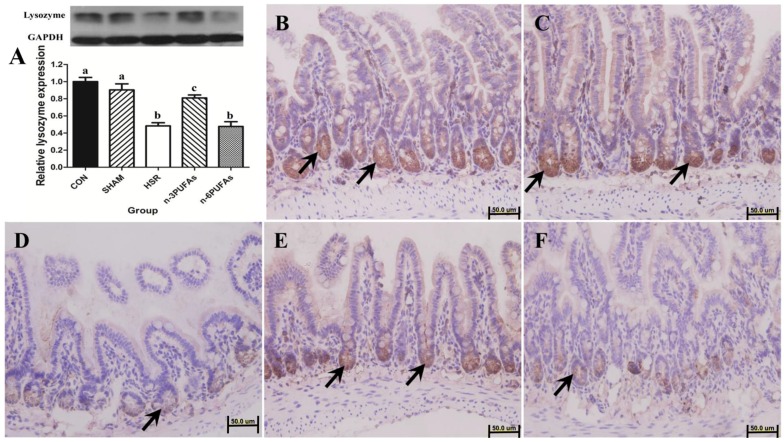
Expression and localization of lysozyme determined by Western blot and immunohistochemistry, respectively, in ileal tissue. (**A**) Western blot showing lysozyme levels in ileal tissue and normalization to GAPDH levels. Columns with different letters are significantly different (*p* < 0.05). CON: control; HSR: hemorrhagic shock resuscitation; PUFAs: polyunsaturated fatty acids; (**B**–**F**) Immunohistochemistry showing lysozyme expression (black arrows) in ileal tissue samples from different groups: (**B**) control group (*n* = 8); (**C**) sham group (*n* = 7); (**D**) HSR group (*n* = 7); (**E**) *n*-3 PUFAs group (*n* = 8); (**F**) *n*-6 PUFAs group (*n* = 7). Original magnification: 40×.

It is known that lysozyme produced and released by the Paneth cells plays an important role in protecting the intestinal mucosa from bacteria invasion [21]. Therefore, the intestinal lysozyme level was assessed. Western blot results showed that the lysozyme level was not different prior to HSR among mice that received *n*-3 PUFAs, *n*-6 PUFAs and saline solution. (Figure S1A). However, lysozyme levels were significantly lower in the HSR group than in the control, sham or *n*-3 PUFA groups (*p* < 0.05, respectively), whereas it was similar to that in the *n*-6 PUFA group (Figure 1A). Despite being lower than in the control and sham groups (*p* < 0.05), lysozyme levels in the *n*-3 PUFAs group indicated that *n*-3 PUFAs could improve lysozyme expression in ileal tissue after HSR injury (*p* < 0.05). Immunohistochemistry showed that lysozyme was localized at the base of the ileal crypts, and its level decreased upon HSR injury. When PUFAs were administrated after resuscitation, immunohistochemistry was consistent with Western blot results. (Figure 1B–F).

### 3.3. mRNA Levels of RegIIIγ and Cryptdin 24 in Ileal Tissue

**Figure 2:**
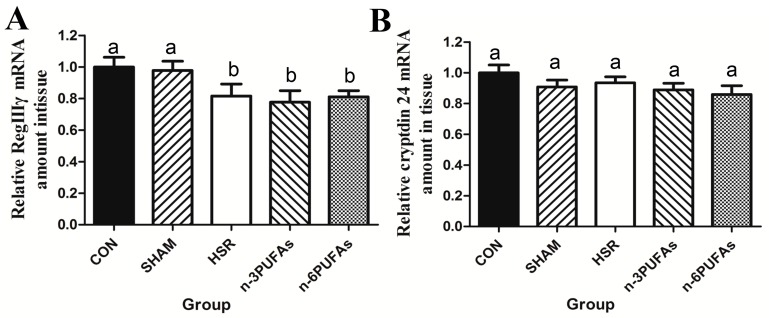
RegIIIγ and cryptdin 24 mRNA levels in ileal tissue, as determined by qPCR. (**A**) RegIIIγ and (**B**) cryptdin 24. Expression levels were normalized to those of GAPDH. Columns with different letters are significantly different (*p* < 0.05). CON: control; HSR: hemorrhagic shock resuscitation; PUFAs: polyunsaturated fatty acids.

It is known that Paneth cells responsible for the production and release of RegIIIγ and cryptdin also play important roles in protecting the intestinal mucosa from bacterial invasion [22]. Therefore, intestinal RegIIIγ and cryptdin 24 levels were assessed. qPCR results showed that both RegIIIγ and cryptdin 24 mRNA levels were not different prior to HSR among mice that received *n*-3 PUFAs, *n*-6 PUFAs and saline solution (Figure S2). Moreover, RegIIIγ mRNA levels in the intestinal mucosa were also not significantly different among the HSR, *n*-3 PUFA and *n*-6 PUFA groups (Figure 2A). Thus, *n*-3 PUFAs and *n*-6 PUFAs failed to restore RegIIIγ mRNA expression after HSR injury (*p* < 0.05). Similarly, cryptdin 24 mRNA levels were not significantly different between the HSR and other groups (Figure 2B).

### 3.4. Mucin 2 Expression and Goblet Cell Count

**Figure 3:**
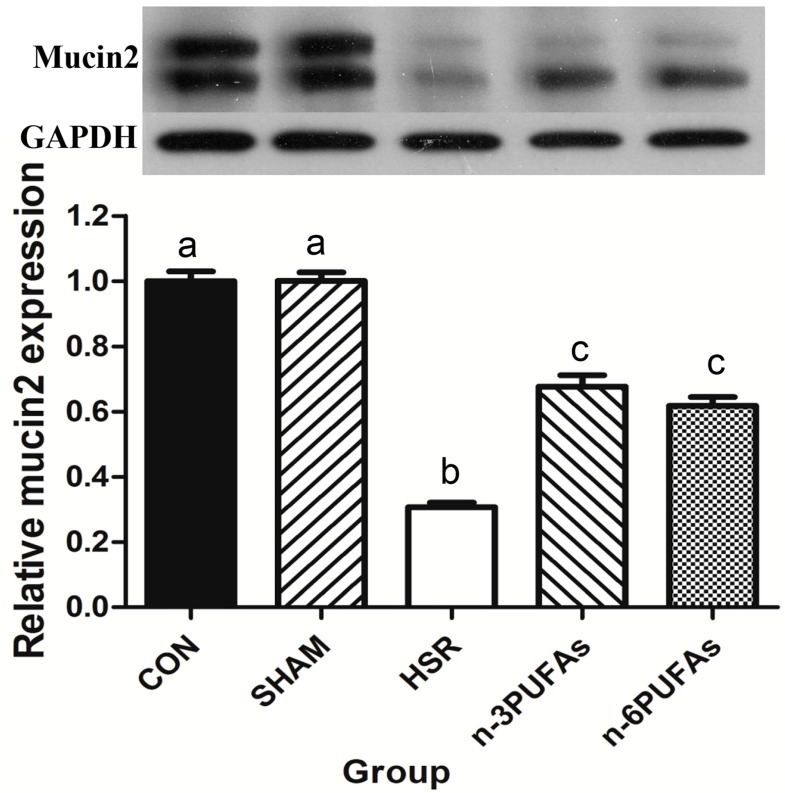
Mucin 2 expression in ileal tissue samples from different groups. Mucin 2 levels were normalized to GAPDH levels. Columns with different letters are significantly different (*p* < 0.05). CON: control; HSR: hemorrhagic shock resuscitation; PUFAs: polyunsaturated fatty acids.

Mucin 2 is produced and secreted continuously by goblet cells, and it is involved in the formation of a mucus barrier to separate intestinal epithelial cells from gut microbiota [11,21]. Therefore, an assessment of intestinal mucin 2 was conducted. Western blot results showed that the mucin 2 level was not different prior to HSR among mice that received *n*-3 PUFAs, *n*-6 PUFAs and saline solution (Figure S1B). Notably, mucin 2 levels were lower in the ileal tissue of the HSR group than in that of the control group (*p* < 0.05) (Figure 3). The addition of *n*-3 PUFAs or *n*-6 PUFAs rescued mucin 2 expression in mice subjected to HSR injury, albeit not completely (*p* < 0.05).

**Figure 4:**
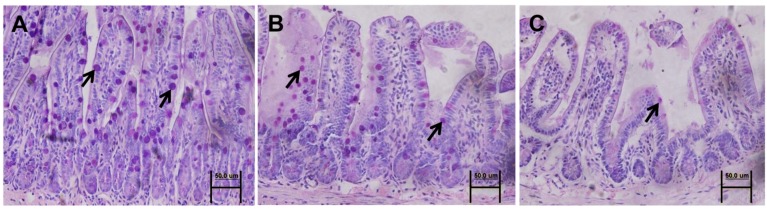

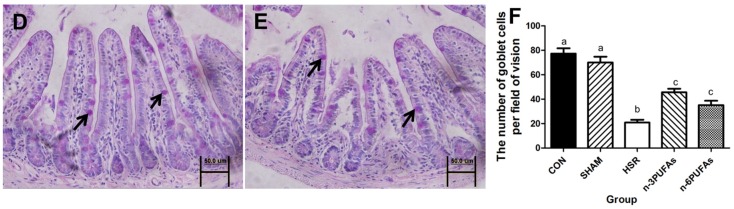
Representative images of ileal tissue samples from the different groups stained with periodic acid-Schiff base. (**A**–**E**) Periodic acid-Schiff base-stained goblet cells in the epithelial layer of the villi (black arrows): (**A**) control group; (**B**) sham group; (**C**) HSR group; (**D**) *n*-3 PUFA group; (**E**) *n*-6 PUFA group. Original magnification: 40×; (**F**) Average number of goblet cells per field. Columns with different letters are significantly different (*p* < 0.05). CON: control; HSR: hemorrhagic shock resuscitation; PUFAs: polyunsaturated fatty acids.

Periodic acid-Schiff staining revealed that the number of mucus-included goblet cells in the HSR group was lower than that in the control group (20.86 ± 6.07 vs. 77.38 ± 12.27; *p* < 0.05). Administration of *n*-3 PUFAs or *n*-6 PUFAs significantly increased the number of mucus-included goblet cells after HSR injury (45.63 ± 8.23 or 35.14 ± 9.44 vs. 20.86 ± 6.07; *p* < 0.05, respectively), albeit incompletely (45.63 ± 8.23 or 35.14 ± 9.44 vs. 77.38 ± 12.27; *p* < 0.05, respectively) (Figure 4A–E). The average number of cells per group is shown in Figure 4F.

### 3.5. Levels of IL-4 and IL-10 in Ileal Tissue

**Figure 5:**
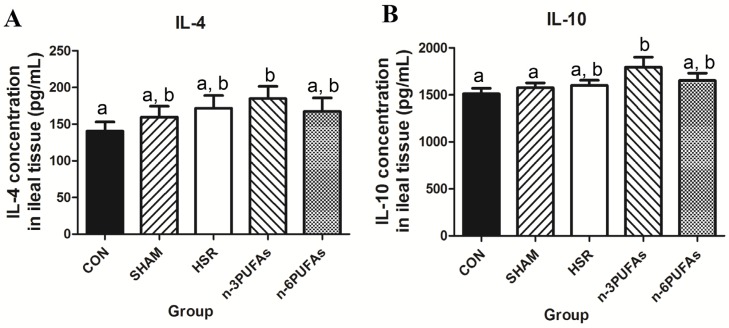
Levels of IL-4 and IL-10 in ileal tissues of different groups. (**A**) IL-4; (**B**) IL-10. Columns with different letters are significantly different (*p* < 0.05). CON: control; HSR: hemorrhagic shock resuscitation; PUFAs: polyunsaturated fatty acids.

In our previous study, decreased tissue levels of mucin 2 and lysozyme were associated with reduced levels of Th2 cytokines, such as IL-4 and IL-10 [21]. Therefore, intestinal tissue levels of IL-4 and IL-10 were assessed. Except in the *n*-3 PUFA group, the IL-4 levels were not significantly higher than those in the control group. Similarly, the IL-10 level in the *n*-3 PUFA group was significantly higher than that in the control and sham groups (1795 ± 301.5 pg/mL vs. 1512 ± 164.6 and 1576 ± 136.8 pg/mL; *p* < 0.05, respectively), whereas the IL-10 level in the HSR and *n*-6 PUFA groups was only marginally higher than that in the sham and control groups (Figure 5A,B).

### 3.6. Relative Abundance of Bacteria in the Intestinal Mucosa

**Figure 6:**
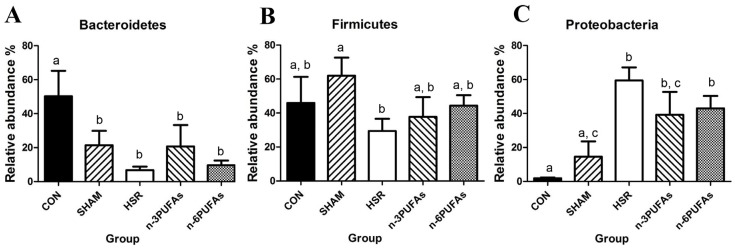
Relative abundance of Bacteroidetes, Firmicutes and Proteobacteria determined by 16S rRNA pyrosequencing of ileal mucosae. (**A**) Bacteroidetes; (**B**) Firmicutes; (**C**) Proteobacteria. Columns with different letters are significantly different (*p* < 0.05). HSR: hemorrhagic shock resuscitation; PUFAs: polyunsaturated fatty acids.

**Figure 7:**
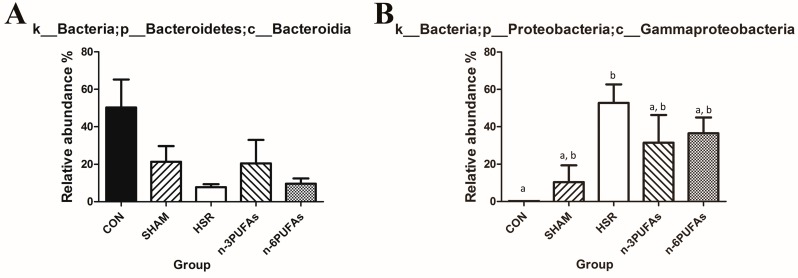
Relative abundance of Bacteroidia and Gammaproteobacteria in the ileal mucosa. (**A**) Bacteroidia; (**B**) Gammaproteobacteria. Columns with different letters are significantly different (*p* < 0.05). CON: control; HSR: hemorrhagic shock resuscitation; PUFAs: polyunsaturated fatty acids.

**Figure 8:**
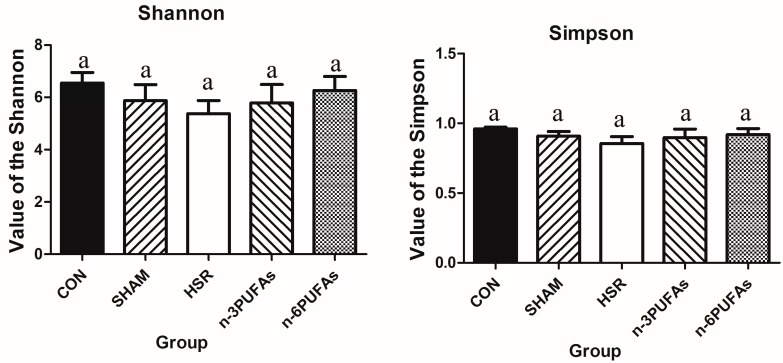
The Shannon index and Simpson index for intestinal microbiota. Columns with different letters indicate significant differences (*p* < 0.05). CON: control; HSR: hemorrhagic shock resuscitation; PUFAs: polyunsaturated fatty acids.

The disturbance of intestinal microbiota is associated with intestinal barrier dysfunction and bacterial translocation. Therefore, we used the bacterial abundance to evaluate the shift of intestinal microbiota. The relative abundance of different bacterial phyla in the intestinal mucosa was derived from 16S rRNA analysis (Figure 6A–C and Figure S3). Compared with controls, the HSR group showed lower relative abundance of Bacteroidetes and greater relative abundance of Proteobacteria (*p* < 0.05). Administration of *n*-3 PUFAs or *n*-6 PUFAs did not significantly increase the relative abundance of Bacteroidetes, partly because of the large variation within groups. The relative abundance of Proteobacteria showed a decreasing trend in the *n*-3 PUFA and *n*-6 PUFA groups compared to the HSR group (*p* > 0.05), but it was still higher than that in the control group (*p* < 0.05). Whereas the relative abundance of Firmicutes was not statistically different among the control, HSR, *n*-3 PUFA and *n*-6 PUFA groups, it was significantly lower in the HSR group than in the sham group (*p* < 0.05). We also evaluated the profile of intestinal microbiota among the five groups at the class level. The heteroscedasticity at the class level was more obvious than that at the phylum level. However, PUFAs indeed restrained the proliferation of Gammaproteobacteria after HSR in mice. (Table S3 and Figure 7). The Shannon index and Simpson index were used to assess the changes in alpha-diversity, and no significant difference was observed among five groups (Figure 8). The statistics for the raw data can be found in the Supplementary Materials (Table S1).

## 4. Discussion

In this study, we established a stable and controlled HSR survival mouse model to study intestinal IRI induced by systemic hypovolemia. First, we assessed the effect of *n*-3 PUFAs on IMII and the intestinal microbiota after HSR. HSR can impair the function of intestinal Paneth and goblet cells. Here, we observed a lower expression of the antimicrobial peptide lysozyme and the glycoprotein mucin 2 in the intestinal tissue of mice after HSR. Although mucin 2 levels were improved by both *n*-3 PUFAs and *n*-6 PUFAs, lysozyme expression was rescued only by *n*-3 PUFAs. The results above indicate that intestinal immuno-barrier function was impaired after HSR and that *n*-3 PUFAs or *n*-6 PUFAs could partially reduce the damage. At the same time, HSR was shown to also affect the intestinal microbiota, which were partially restored upon addition of *n*-3 PUFAs and, to a lesser extent, *n*-6 PUFAs. Moreover, *n*-3 PUFAs also increased the expression of IL-4 and IL-10 in ileal tissue.

HSR or ischemia-reperfusion could damage the intestinal epithelial tight junction, as confirmed by our previous studies [6,7]. Increased intestinal mucosal permeability after surgery might lead to bacterial translocation [9]. Indeed, the higher bacterial counts in MLN were in accordance with previous studies [23]. The inflammatory response and the activated immune system may help prevent bacterial translocation from the MLN to the liver and spleen, as confirmed here by the lack of positive bacterial colonies in these two organs. Although one specific set of culturing conditions may not completely reflect overall bacterial translocation, the results of the bacterial culture partly indicate that the intestinal barrier function was impaired by HSR. Therefore, the bacterial translocation attributed to damage to the IMII or intestinal mechanical barrier after HSR needs further investigation. A recent study described apparent Paneth cell apoptosis in tissue exposed to 45–60 min of ischemia with 30 min of reperfusion [13]. In a different study, goblet cells appeared to release their contents into the lumen after 60 min of ischemia, and the mucus-depleted goblet cells were dominant after 60 min of reperfusion [11]. The present study revealed that 90 min of hemorrhagic shock followed by resuscitation decreased the levels of lysozyme and mucin 2, consistent with previous reports in different mouse models of IR [11,13,24]. Consequently, decreased lysozyme and mucin 2 levels in the intestinal mucosa may contribute to increased bacterial translocation after HSR [25].

It should be noted that *n*-3 PUFAs were previously reported to help maintain the levels of antibacterial peptides in Paneth cells in a food allergy mouse model [26]. Here, we observed that *n*-3 PUFAs partially restored the protein expression of lysozyme and mucin 2 in the injured intestinal mucosae, in line with *n*-3 PUFAs reducing bacterial translocation. At the same time, *n*-3 PUFAs were found to stimulate the secretion of T Helper 2 cytokines IL-4 and IL-10 in the intestinal mucosa. This is consistent with a previous study, which showed that *n*-3 PUFAs increased the levels of IL-10 in mice subjected to *Citrobacter rodentium* infection [27]. Increased levels of anti-inflammatory cytokines confirmed that *n*-3 PUFAs could alleviate the systemic inflammatory response induced by injury [28,29,30]. Furthermore, based on previous studies, *n*-3 PUFAs may also reverse the downregulation of lysozyme and mucin 2 after HSR [21,31,32]. The metabolites of *n*-6 PUFAs can modulate the function of group 2 innate lymphoid cells, which are involved in maintaining the level of tissue mucin 2 [33,34]. However, it remains to be determined why *n*-6 PUFAs, but not lysozyme, enhanced the expression of mucin 2, but not lysozyme, IL-4, or IL-10. In addition, *n*-6 PUFAs also ameliorated bacterial translocation after HSR in MLN, possibly through increased mucin 2 expression [25]. It should be noted, however, that exogenous addition of *n*-3 PUFAs or *n*-6 PUFAs failed to revert the low expression of the antibacterial lectin RegIIIγ. Although lysozyme and lectin RegIIIγ were both generated in Paneth cells, *n*-3 PUFAs increased the tissue level of lysozyme, but not lectin RegIIIγ after HSR. The potential mechanisms underlying such effects require further investigation. Many earlier studies demonstrated the greater anti-inflammatory potential of *n*-3 PUFAs compared with that of *n*-6 PUFAs [6,7,35]. However, this notion has been challenged by later research. Some studies reported that arachidonic acid, one of *n*-6 PUFAs, was not only a precursor of pro-inflammatory bioactive lipids, but also a member of the potent anti-inflammatory mediators known as lipoxins [36,37,38,39,40]. Several reports have indicated a protective role for lipoxins or stable lipoxin analogues in ischemia-reperfusion [41,42,43]. Gobbetti et al. (2015) reported that high levels of dietary *n*-6 PUFAs, but not *n*-3 PUFAs, provided significant protection against IRI. In that study, nine weeks of dietary PUFA intake were found to directly influence the intestinal microbial communities, and the subsequent formation of bacterial metabolites might play important roles in this process [41]. In addition, *n*-3 and *n*-6 PUFAs are natural ligands of the G-protein coupled receptor 120, which has been demonstrated to inhibit NF-κB signaling upon binding to Toll-like receptor 4 in macrophages and adipocytes [44,45]. Therefore, the effects of *n*-3 PUFAs versus *n*-6 PUFAs on intestinal immuno-barrier function and the potential mechanisms involved require further elucidation. Taken together, our results suggest that *n*-3 PUFAs could partially improve the function of Paneth cells after HSR injury, whereas both *n*-3 PUFAs and *n*-6 PUFAs could alleviate IRI in goblet cells after HSR.

The intestinal microbial communities also changed as the antimicrobial peptide AMP and MUC2 changed. Several studies have demonstrated cross-talk between AMP and intestinal bacteria [12,46]. Bacteroidetes and Firmicutes are the two dominant commensal bacterial phyla, whereas Proteobacteria include pathogenic bacteria, such as *Salmonella* and *Vibrio cholerae* in the intestine. Compared with that in the control group, the relative abundance of Bacteroidetes in the other four groups was lower, whereas that of Proteobacteria was higher. Except in the sham group, the abundance of Firmicutes did not change significantly. HSR-induced damage to Paneth and goblet cells may contribute to the disturbed intestinal microbiota. Previous studies indicated that ischemia-reperfusion could promote the release of AMP and mucin 2 into the intestinal tube from surrounding tissue [11,13]. The sharp increase in AMP and mucin 2 in the intestinal tube after HSR or surgical injury might be responsible for the observed reduced numbers of Bacteroidetes, but not Firmicutes. A rapid recovery of IMII in the sham group, but not in the groups subjected to HSR, likely inhibited the proliferation of Proteobacteria, while indirectly augmenting the numbers of Firmicutes. Consequently, as the number of Bacteroidetes was smaller, changes to the intestinal bacterial composition were observed in mice recovering from injuries of different extents. In contrast, the reduced relative abundance of Proteobacteria, especially Gammaproteobacteria, upon administration of *n*-3 PUFAs and *n*-6 PUFAs might be associated with the improved function of Paneth and goblet cells. Specifically, we observed that *n*-6 PUFAs increased mucin 2 levels, which coincided with the lower relative abundance of Gammaproteobacteria compared to the HSR group. Therefore, we speculated that lower mucin 2 levels may be associated with the increased relative abundance of Gammaproteobacteria, as proposed by a recent study [11]. The resistance of Bacteroidetes to high levels of inflammation-induced AMP would lead to more Bacteroidetes colonizing the mucus than Proteobacteria [12]. It should be noted that only five mice per group except in the HSR group (six individuals) were subjected to 16S rRNA pyrosequencing, and the large variation within groups might have led to some negative results. Thus, the presently reported results of the 16S rRNA analysis should be treated with caution because of these limitations.

## 5. Conclusions

In conclusion, a hemorrhagic shock resuscitation mouse model was used to simulate the intestinal ischemia-reperfusion injury induced by systemic hypovolemia. This work supports our hypothesis that HSR suppresses the function of Paneth cells through decreased expression of lysozyme and RegIIIγ and reduces the function and number of goblet cells. As a result, the structure of the intestinal microbiome was also disturbed, leading to fewer Bacteroidetes and more Proteobacteria. *n*-3 PUFAs exerted a partially protective role on innate immunity of the intestinal mucosa by restoring the expression of lysozyme and mucin 2, increasing the IL-4 level and helping to stabilize the intestinal microbiota. Although they were less effective, *n*-6 PUFAs also rescued mucin 2 expression in mice after HSR. This study may provide a reliable theoretical basis for the beneficial use of *n*-3 PUFAs to improve intestinal barrier function in post-traumatic patients who have experienced ischemia-reperfusion injury. It may also provide guidance towards more effective nutritional support for surgical patients and, thus, optimize the treatment.

## Figures and Tables

**Table 1:** Primers used for qPCR.

Target Gene	Primer	Sequence (5′–3′)
GAPDH	Forward Primer	AGGCCGGTGCTGAGTATGTC
	Reverse Primer	TGCCTGCTTCACCACCTTCT
Cryptdin 24	Forward Primer	TGAAGACACTAATCCTCCTCTCTGC
	Reverse Primer	GCTCCTCAGTTTTAGTCTCTTCATCTGTA
RegIIIγ	Forward Primer	GTATGATGCAGATATGGCCTG
	Reverse Primer	ATATTGGCCACTGTTACCAC

**Table 2:** Colony counts of tissue bacterial cultures.

	Control	Sham	HSR	*n*-3 PUFAs	*n*-6 PUFAs
Liver	0 ^a^	0 ^a^	200 ^a^	0 ^a^	0 ^a^
Spleen	0 ^a^	0 ^a^	0 ^a^	0 ^a^	0 ^a^
MLN	0 ^a^	0 ^a^	4000 ^b^	550 ^c^	900 ^c^

Data are expressed in CFU/g and presented as the median. Columns with different letters are significantly different (*p* < 0.05). CFU: colony-forming unit; HSR: hemorrhagic shock resuscitation; PUFAs: polyunsaturated fatty acids; MLN, mesenteric lymph nodes.

## Supplementary Materials

The following are available online at http://www.mdpi.com/2072-6643/8/10/609/s1: Table S1: Raw data for bacteria in the intestinal mucosa; Table S2: Positive rates of bacterial translocation in tissue bacterial cultures; Table S3: Intestinal microbiota were analyzed by 16S rDNA pyrosequencing at the level of class. Figure S1: Lysozyme and mucin 2 expression in ileal tissue samples from different groups prior to HSR; Figure S2: RegIIIγ and cryptdin 24 mRNA level in ileal tissue samples from different groups prior to HSR; Figure S3: Relative abundance of bacterial phyla in the ileum mucosa. CON: control; HSR: hemorrhagic shock resuscitation; PUFAs: polyunsaturated fatty acids.

## Abbreviations

The following abbreviations are used in this manuscript:

PUFAs	Polyunsaturated fatty acids
HSR	Hemorrhagic shock resuscitation
IRI	Ischemia-reperfusion injury
IMII	Intestinal mucosa innate immunity
CON	Control
MAP	Mean arterial pressure
PBS	Phosphate-buffered saline
CFUs	Colony-forming units
ELISA	Enzyme-linked immunosorbent assay
GAPDH	Glyceraldehyde-3-phosphate dehydrogenase
