# Supplementary material for: Effects of n-3 PUFAs on Intestinal Mucosa Innate Immunity and Intestinal Microbiota in Mice after Hemorrhagic Shock Resuscitation
Source: Nutrients. 2016 Sep 29;8(10):609. doi: 10.3390/nu8100609 (PMC5083997; doi:10.3390/nu8100609)
Supplement: Supplementary file 1 [file nutrients-08-00609-s001.docx]

Supplementary Materials: Effects of *n*-3 PUFAs
on Intestinal Mucosa Innate Immunity and
Intestinal Microbiota in Mice after Hemorrhagic Shock Resuscitation

Feng Tian, Xuejin Gao, Li Zhang, Xinying Wang, Xiao Wan, Tingting Jiang, Chao Wu,
Jingcheng Bi and Qiucheng Lei


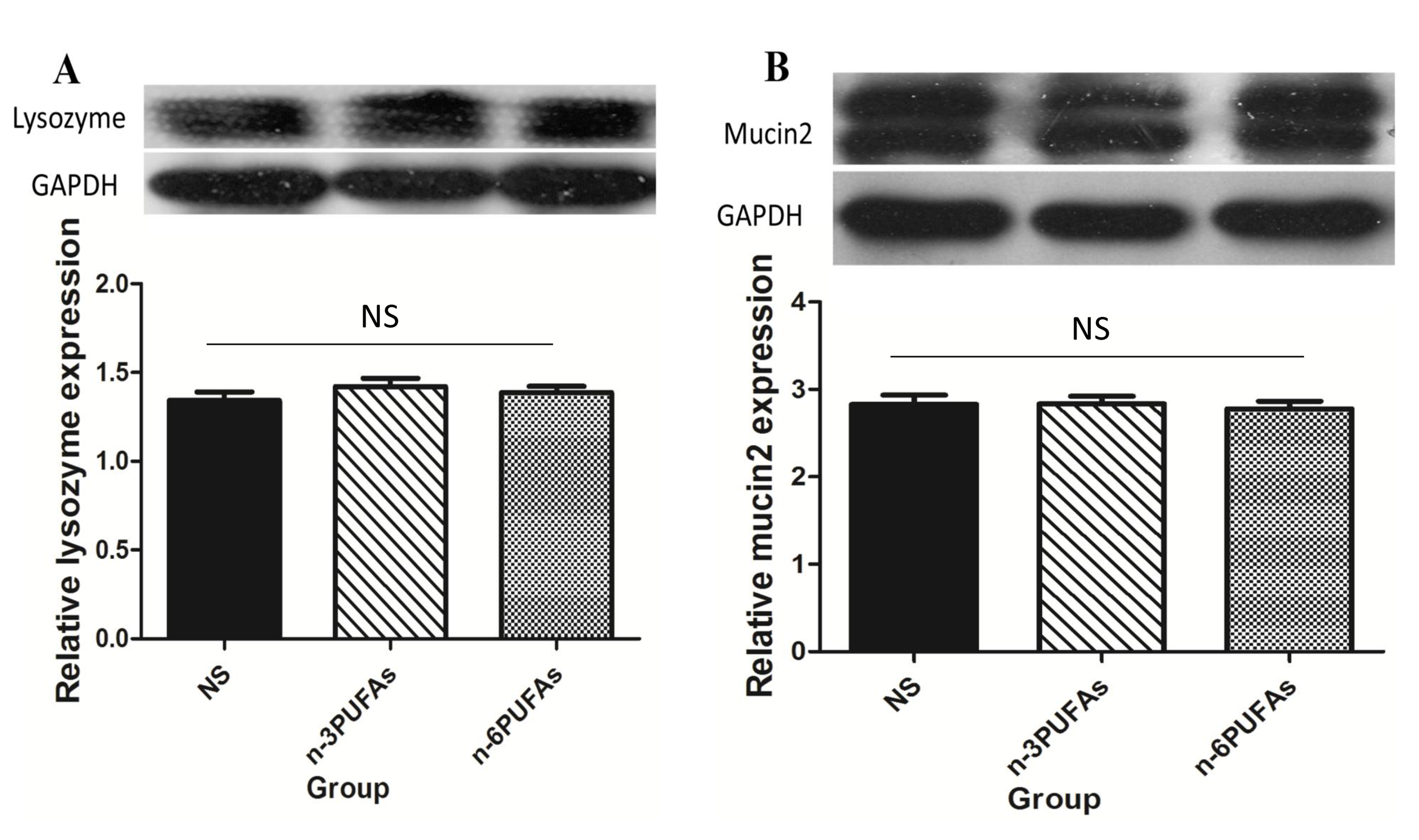


**Figure S1.** Lysozyme and mucin 2 expression in ileal tissue samples from different groups prior
to HSR.


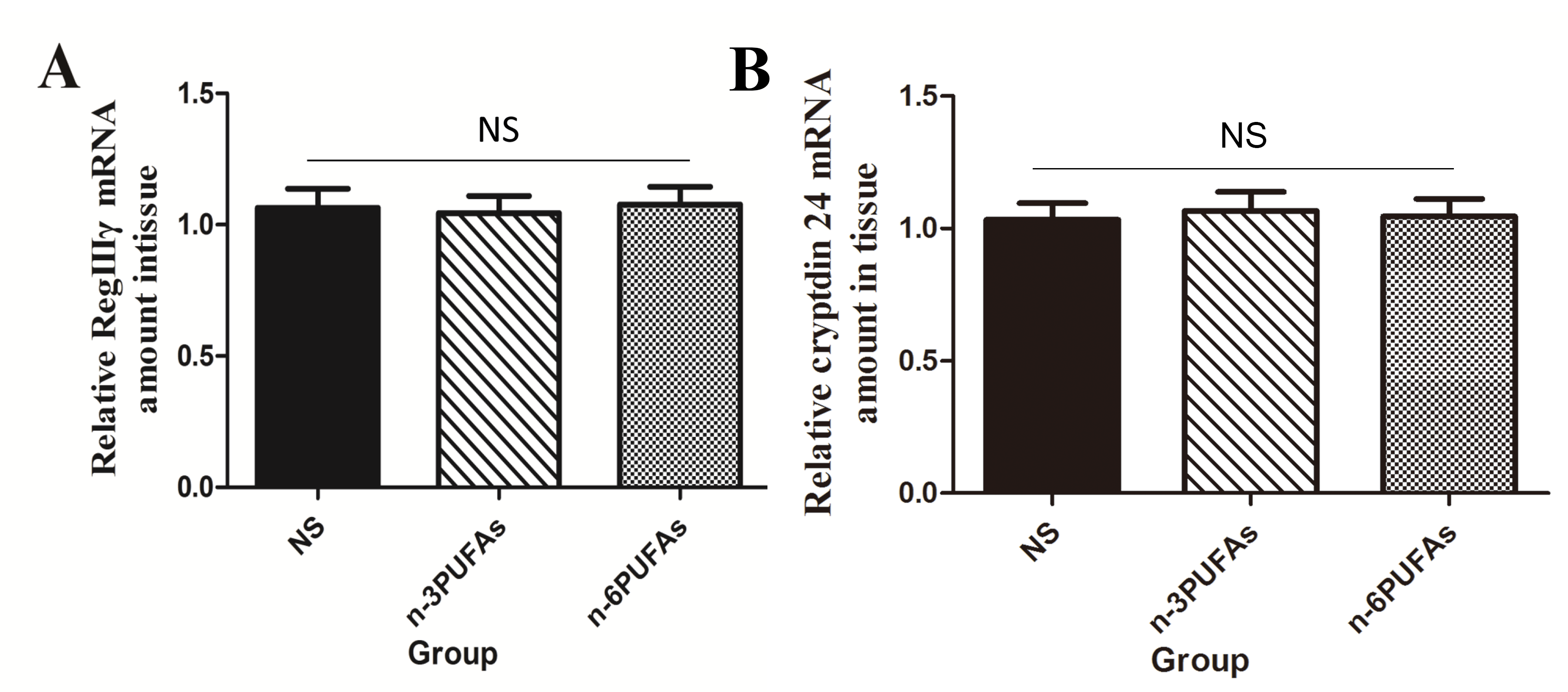


**Figure S2.** RegIIIγ and cryptdin 24 mRNA level in ileal tissue samples from different groups prior
to HSR.


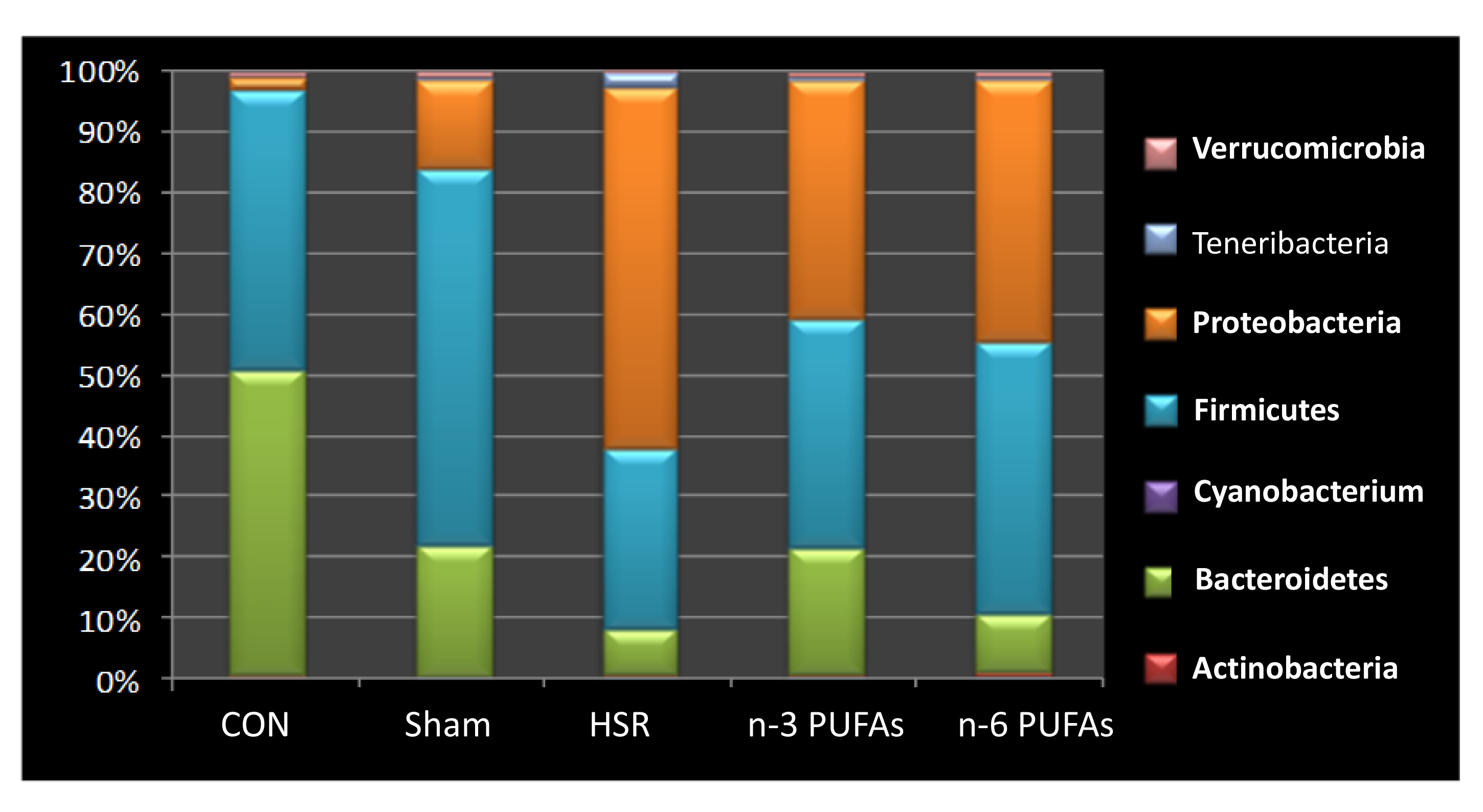


**Figure S3.** Relative abundance of bacterial phyla in the ileum mucosa. CON: control; HSR: hemorrhagic shock resuscitation; PUFAs: polyunsaturated fatty acids.

**Table S1.** Statistics of Raw Data.

| **Groups** | **Samples** | **Total Length** | **Average Length** | **Number of Reads** | **Number of OTUs** | **Taxonomy** | **Other** | **Actinobacteria** | **Bacteroidetes** | **Cyanobacteria** | **Firmicutes** | **Proteobacteria** | **Tenericutes** | **Verrucomicrobia** |
| --- | --- | --- | --- | --- | --- | --- | --- | --- | --- | --- | --- | --- | --- | --- |
| CON | 1 | 63052725 | 290.89 | 105413 | 487 | **%** | **0.30%** | **1.20%** | **81.90%** | **0.00%** | **12.30%** | **1.70%** | **0.00%** | **2.60%** |
|  | 2 | 71892548 | 294.78 | 121361 | 633 | **%** | **0.40%** | **0.90%** | **82.50%** | **0.00%** | **14.00%** | **1.50%** | **0.00%** | **0.60%** |
|  | 3 | 86575013 | 269.68 | 122558 | 834 | **%** | **0.20%** | **0.10%** | **22.70%** | **0.00%** | **76.00%** | **0.70%** | **0.20%** | **0.00%** |
|  | 4 | 76445720 | 274.61 | 119926 | 850 | **%** | **0.20%** | **0.50%** | **54.60%** | **0.00%** | **41.60%** | **2.70%** | **0.00%** | **0.20%** |
|  | 5 | 81399654 | 275.31 | 102926 | 729 | **%** | **0.10%** | **0.00%** | **9.90%** | **0.10%** | **86.00%** | **3.00%** | **0.60%** | **0.20%** |
| Sham | 6 | 62866266 | 296.17 | 98424 | 497 | **%** | **0.50%** | **0.40%** | **47.60%** | **0.00%** | **46.80%** | **4.70%** | **0.00%** | **0.00%** |
|  | 7 | 83037080 | 270.46 | 112986 | 849 | **%** | **0.30%** | **0.00%** | **8.90%** | **0.00%** | **89.20%** | **1.40%** | **0.10%** | **0.10%** |
|  | 8 | 97327368 | 265.02 | 146530 | 865 | **%** | **0.30%** | **0.00%** | **32.50%** | **0.00%** | **61.10%** | **1.10%** | **0.40%** | **4.70%** |
|  | 9 | 41203670 | 283.99 | 72472 | 347 | **%** | **1.50%** | **0.00%** | **0.10%** | **0.30%** | **81.10%** | **17.00%** | **0.00%** | **0.00%** |
|  | 10 | 55281460 | 287.03 | 102315 | 923 | **%** | **0.30%** | **0.20%** | **18.20%** | **0.00%** | **32.10%** | **48.70%** | **0.20%** | **0.40%** |
| HSR | 11 | 46665104 | 295.11 | 99785 | 727 | **%** | **0.50%** | **0.00%** | **13.00%** | **0.10%** | **11.30%** | **74.50%** | **0.20%** | **0.30%** |
|  | 12 | 36223900 | 277.58 | 63267 | 709 | **%** | **0.40%** | **0.20%** | **3.80%** | **0.00%** | **29.20%** | **65.80%** | **0.60%** | **0.00%** |
|  | 13 | 42912879 | 276.59 | 79836 | 697 | **%** | **0.70%** | **0.80%** | **7.10%** | **0.00%** | **35.40%** | **44.60%** | **11.40%** | **0.00%** |
|  | 14 | 199892267 | 290.90 | 484192 | 793 | **%** | **0.90%** | **0.10%** | **4.50%** | **0.00%** | **49.80%** | **44.00%** | **0.80%** | **0.00%** |
|  | 15 | 277431224 | 302.32 | 647077 | 1067 | **%** | **0.20%** | **0.40%** | **12.20%** | **0.10%** | **44.80%** | **41.00%** | **0.60%** | **0.60%** |
|  | 16 | 37901582 | 299.57 | 87884 | 607 | **%** | **0.30%** | **0.10%** | **6.10%** | **0.10%** | **6.10%** | **86.60%** | **0.60%** | **0.00%** |
| *n*-3 PUFAs | 17 | 78554581 | 294.28 | 123297 | 631 | **%** | **0.70%** | **0.60%** | **66.80%** | **0.00%** | **10.50%** | **18.70%** | **0.00%** | **2.70%** |
|  | 18 | 62068779 | 285.16 | 92856 | 885 | **%** | **0.30%** | **0.40%** | **29.00%** | **0.20%** | **53.60%** | **14.30%** | **2.10%** | **0.20%** |
|  | 19 | 259689151 | 305.13 | 528572 | 850 | **%** | **0.40%** | **0.30%** | **2.00%** | **0.00%** | **49.60%** | **47.10%** | **0.50%** | **0.10%** |
|  | 20 | 318985239 | 300.02 | 792287 | 829 | **%** | **0.20%** | **0.20%** | **1.50%** | **0.00%** | **9.60%** | **88.00%** | **0.40%** | **0.20%** |
|  | 21 | 266520892 | 297.78 | 619464 | 1068 | **%** | **1.20%** | **0.60%** | **4.10%** | **0.00%** | **65.40%** | **28.10%** | **0.50%** | **0.20%** |
| *n*-6 PUFAs | 22 | 257349936 | 304.26 | 535098 | 946 | **%** | **0.70%** | **0.80%** | **5.00%** | **0.00%** | **20.70%** | **69.80%** | **1.10%** | **2.00%** |
|  | 23 | 312192992 | 301.71 | 713002 | 1093 | **%** | **0.30%** | **2.30%** | **18.40%** | **0.10%** | **50.80%** | **25.40%** | **0.40%** | **2.30%** |
|  | 24 | 213185174 | 293.36 | 500018 | 914 | **%** | **0.90%** | **0.90%** | **7.30%** | **0.00%** | **53.90%** | **36.70%** | **0.10%** | **0.20%** |
|  | 25 | 40962015 | 266.24 | 66147 | 722 | **%** | **0.70%** | **0.20%** | **3.70%** | **0.00%** | **52.20%** | **43.00%** | **0.20%** | **0.00%** |
|  | 26 | 70459052 | 284.87 | 118827 | 831 | **%** | **0.40%** | **0.10%** | **13.80%** | **0.10%** | **44.30%** | **40.40%** | **0.80%** | **0.10%** |

**Table S2.** Positive rates of bacterial translocation in tissue bacterial cultures.

|  | **Control** | **Sham** | **HSR** | ***n*-3 PUFAs** | ***n*-6 PUFAs** |
| --- | --- | --- | --- | --- | --- |
| Liver | 1/8 | 1/7 | 4/7 | 2/8 | 2/7 |
| Spleen | 0/8 | 1/7 | 3/7 | 1/8 | 2/7 |
| MLN | 2/8 ^#^ | 3/7 | 6/7 * | 7/8 * | 6/7 * |

Rates are presented as ratios of the positive number of bacterial cultures/total number of bacterial cultures. *: *p* < 0.05 vs. CON; ^#^: *p* < 0.05 vs. HSR. MLN: Mesenteric lymph node; HSR: Hemorrhagic shock resuscitation; PUFAs: Polyunsaturated fatty acids.

**Table S3.** Intestinal microbiota was analyzed by 16S rDNA pyrosequencing at levels of class.

| **Taxonomy-Class** | **Homogeneity Test of Variances** | **One Way ANOVA** | |
| --- | --- | --- | --- |
|  | ***p*** | ***F*** | ***p*** |
| Unassigned; Other; Other | 0.23 | 1.32 | 0.29 |
| k__Bacteria; p__Actinobacteria; Other | <0.01 | 0.81 | 0.53 |
| k__Bacteria; p__Actinobacteria; c__Actinobacteria | <0.01 | 2.36 | 0.09 |
| k__Bacteria; p__Actinobacteria; c__Coriobacteriia | 0.01 | 2.00 | 0.13 |
| k__Bacteria; p__Bacteroidetes; c__Bacteroidia | <0.01 | 3.42 | 0.03 |
| k__Bacteria; p__Bacteroidetes; c__Flavobacteriia | <0.01 | 0.98 | 0.44 |
| k__Bacteria; p__Bacteroidetes; c__Sphingobacteriia | <0.01 | 1.33 | 0.29 |
| k__Bacteria; p__Cyanobacteria; c__4C0d-2 | <0.01 | 1.28 | 0.31 |
| k__Bacteria; p__Cyanobacteria; c__Chloroplast | 0.02 | 0.54 | 0.71 |
| k__Bacteria; p__Firmicutes; Other | <0.01 | 1.82 | 0.16 |
| k__Bacteria; p__Firmicutes; c__Bacilli | <0.01 | 1.87 | 0.15 |
| k__Bacteria; p__Firmicutes; c__Clostridia | 0.04 | 0.65 | 0.63 |
| k__Bacteria; p__Firmicutes; c__Erysipelotrichi | 0.02 | 1.99 | 0.13 |
| k__Bacteria; p__Proteobacteria; c__Alphaproteobacteria | <0.01 | 1.03 | 0.41 |
| k__Bacteria; p__Proteobacteria; c__Betaproteobacteria | 0.29 | 1.10 | 0.38 |
| k__Bacteria; p__Proteobacteria; c__Deltaproteobacteria | 0.01 | 0.78 | 0.55 |
| k__Bacteria; p__Proteobacteria; c__Epsilonproteobacteria | 0.12 | 0.81 | 0.53 |
| k__Bacteria; p__Proteobacteria; c__Gammaproteobacteria | 0.02 | 4.77 | 0.01 |
| k__Bacteria; p__Tenericutes; c__Mollicutes | 0.01 | 1.00 | 0.43 |
| k__Bacteria; p__Verrucomicrobia; c__Opitutae | <0.01 | 0.96 | 0.45 |
| k__Bacteria; p__Verrucomicrobia; c__Verrucomicrobiae | 0.06 | 0.44 | 0.78 |
